# Beyond Genetic Factors in Familial Amyloidotic Polyneuropathy: Protein Glycation and the Loss of Fibrinogen's Chaperone Activity

**DOI:** 10.1371/journal.pone.0024850

**Published:** 2011-10-28

**Authors:** Gonçalo da Costa, Ricardo A. Gomes, Ana Guerreiro, Élia Mateus, Estela Monteiro, Eduardo Barroso, Ana V. Coelho, Ana Ponces Freire, Carlos Cordeiro

**Affiliations:** 1 Centro de Química e Bioquímica, Departamento de Química e Bioquímica, Faculdade de Ciências da Universidade de Lisboa, Lisboa, Portugal; 2 Instituto de Tecnologia Química e Biológica, Oeiras, Portugal; 3 Unidade de Transplantação Hepática, Hospital Curry Cabral, Lisboa, Portugal; 4 Departamento de Química da Universidade de Évora, Évora, Portugal; Purdue University, United States of America

## Abstract

Familial amyloidotic polyneuropathy (FAP) is a systemic conformational disease characterized by extracellular amyloid fibril formation from plasma transthyretin (TTR). This is a crippling, fatal disease for which liver transplantation is the only effective therapy. More than 80 TTR point mutations are associated with amyloidotic diseases and the most widely accepted disease model relates TTR tetramer instability with TTR point mutations. However, this model fails to explain two observations. First, native TTR also forms amyloid in systemic senile amyloidosis, a geriatric disease. Second, age at disease onset varies by decades for patients bearing the same mutation and some mutation carrier individuals are asymptomatic throughout their lives. Hence, mutations only accelerate the process and non-genetic factors must play a key role in the molecular mechanisms of disease. One of these factors is protein glycation, previously associated with conformational diseases like Alzheimer's and Parkinson's. The glycation hypothesis in FAP is supported by our previous discovery of methylglyoxal-derived glycation of amyloid fibrils in FAP patients. Here we show that plasma proteins are differentially glycated by methylglyoxal in FAP patients and that fibrinogen is the main glycation target. Moreover, we also found that fibrinogen interacts with TTR in plasma. Fibrinogen has chaperone activity which is compromised upon glycation by methylglyoxal. Hence, we propose that methylglyoxal glycation hampers the chaperone activity of fibrinogen, rendering TTR more prone to aggregation, amyloid formation and ultimately, disease.

## Introduction

Familial amyloidotic polyneuropathy (FAP) is an autosomic dominant neurodegenerative disease characterized by the formation of amyloid fibril deposits, mainly composed of transthyretin (TTR), in different organs and tissues [1], [2]. It is a progressive and crippling disease that ultimately leads to death. FAP is associated with point mutations in TTR, a homotetrameric protein mainly produced in the liver and found in the plasma, cerebrospinal fluid and saliva. Over 80 TTR point mutations are related to TTR amyloidogenic behavior and amyloidotic diseases leading to systemic amyloid fibril formation with the characteristic β-sheet cross structure commonly found in several other neurodegenerative disorders such as Alzheimer and Parkinson [3]. Since TTR is mainly produced by the liver, the only effective therapeutic option for FAP is the orthotopic liver transplantation (OLT) from cadaveric donors. This method was first validated in 1990 in Sweden since OLT leads to the clearance of V30M TTR from the plasma of the FAP transplanted recipient [4]. To obviate the shortage of livers available for transplantation, domino liver transplantation (DLT) was recently introduced in which a liver from a FAP patient is transplanted to a patient with liver failure. DLT introduces mutated TTR variants in circulation, increasing the risk of FAP development [5]. Currently, the main hypothesis for FAP pathogenesis considers that point mutations cause TTR tetramer instability favoring its dissociation to non-native monomeric species with the ability to self-associate [6]. These soluble monomers tends to aggregate and evolve to insoluble multimeric forms leading to amyloid fibrils with the characteristic β-sheet cross structure [6]. However, this model fails to explain two crucial aspects of TTR amyloid formation and pathogenesis. First, mutations are not required for TTR amyloid formation. Indeed, non-mutated TTR also forms amyloid deposits in systemic senile amyloidosis, a crippling disease in later life [7]. Also, wild type TTR continues to accumulate into amyloid deposits even after liver transplantation [8]. In fact, after liver transplantation, FAP patients present a shorter life-span than expected which may be associated with progression of neuropathy due to continued deposition of non-mutated TTR in amyloid form [8]. Moreover, a considerable number of TTR mutation carriers are asymptomatic throughout their lives [9]. Thus, point mutations only modify the intrinsic amyloidogenic nature of TTR and are not absolute predictors of amyloid formation or disease development. Second, time of disease onset varies by decades for different patients bearing the same mutation [10] and also the genetic trait frequency in different areas is not correlated with the number of known cases [11]. Furthermore, discordant symptoms in homozygote twins were observed: while one of the twins underwent liver transplantation, the other remained healthy for at least 8 years after his brother's disease onset [12]. It is also noteworthy that the homozygous carriers of the amyloidogenic TTR variant V30M do not develop a more aggressive disease form than heterozygous ones [13], [14], [15]. The most likely explanation for these observations is the involvement of non-genetic factors in FAP onset and disease progression. Uncovering the nature of these non-genetic factors and the molecular mechanisms of their actions is likely to have a major impact on our knowledge of the molecular mechanisms of other conformational diseases. The two most important non-genetic factors that may directly affect protein structure and function are protein-protein interactions and post-translational modifications. In the first one, it is remarkable that TTR does not form amyloid deposits in the cerebrospinal fluid. TTR interacts in the cerebrospinal fluid with different interactors than in the plasma, like thyroxine T4, which prevents TTR amyloid fibril formation [16]. Concerning the second, among a legion of known post-translational modifications, glycation is the common link in amyloidotic pathologies, being found in Alzheimer, Parkinson and also in FAP [17]. One of the most powerful glycation agents *in vivo* is methylglyoxal formed in all living cells from dihydroxyacetone phosphate and D-glyceraldehyde 3-phosphate, as a non-enzymatic glycolysis by-product [18]. Methylglyoxal irreversibly modifies both lysine and arginine residues in proteins forming advanced glycation end products, termed MAGE (methylglyoxal-derived advanced glycation end products) [18]. Arginine-derived MAGE appear to be more relevant considering the existence of specific receptors for hydroimidazolones [19]. Argpyrimidine [Nδ-(5-hydroxy-4,6-dimethylpyrimidin- 2-yl)-L-ornithine] is another arginine MAGE [20] abundantly found *in vivo* [21], [22]. Methylglyoxal glycation was found in several conformational pathologies such as Alzheimer [23], [24], [25], [26], dialysis-related amyloidosis [27] Parkinson [28], prion diseases [29], hemodialysis-related A_2M amyloidosis, [30], murine ApoAII amyloidosis [31]. We previously reported that amyloid fibrils extracted from FAP patients are glycated by methylglyoxal, bringing further support to the hypothesis that methylglyoxal-derived protein glycation is involved in conformational diseases [22].

In the present study, we found that FAP patients present higher levels of argpyrimidine-modified proteins in human plasma. Moreover, we discovered that fibrinogen is a specific glycation target with an increased glycation in FAP patients and is one of the main TTR interacting proteins in plasma. Upon glycation by methylglyoxal, we observed a significant reduction of the fibrinogen chaperone activity *in vitro*. Considering that we observed that fibrinogen prevents plasma TTR thermal-induced protein aggregation, we created a new molecular model for TTR amyloidogenesis *in vivo*. Briefly, in FAP, increased glycation of fibrinogen reduces its chaperone activity, reducing TTR tetramer stability and triggering the pathway to aggregation, amyloid formation and disease.

## Materials and Methods

### Ethics Statement

The ethics board of the Hospital de Curry Cabral approved all procedures regarding human blood collection. All subjects involved gave informed written consent and the protocol was approved according to EEC ethic rules and the Declaration of Helsinki.

### Human plasma Collection

Blood samples from control individuals, FAP patients (four females each group, age range 26–33 years) and transplanted individuals (males age range 24–41 years for OLT (orthotopic liver transplantation) and age range 43–58 years for DLT (domino liver transplantation) were collected to citrate containing tubes, centrifuged at 1,800 *g* for 5 min at 4°C and the collected plasma was immediately frozen at −80°C until further analysis. Blood was collected by venous puncture to citrate containing tubes.

### Poliacrylamide Gel Electrophoresis

Plasma proteins were separated by sodium dodecyl sulfate polyacrilamide gel electrophoresis (12% SDS-PAGE), in mini-gel format (7×7 cm Tetra system from Bio-Rad). Twenty micrograms of plasma protein were used per lane. Protein concentration was determined by the Bradford protein assay, using bovine serum albumin as standard. Samples were diluted 10 fold in MilliQ water and mixed with reduction buffer (62.5 mM Tris-HCl, pH 6.8, 20% (v/v) glycerol, 2% (w/v) SDS, 5% (v/v) β-mercaptoetanol). Prior to electrophoresis, samples were heated at 100°C for 5 min. Protein bands were stained with Coomassie Brilliant Blue R-250.

### Two Dimensional Electrophoresis

Samples containing 15 or 250 µg of plasma protein were used for IEF (isoelectric focusing) using immobiline dry strips of 7 or 13 cm, respectively, with a non linear pH gradient from 3 to 11 [32] (Amersham Biosciences). After IEF separation, proteins were reduced and alkylated in the dry strip. Second dimension SDS-PAGE was performed using 12% SDS-PAGE. Protein spots were stained with Coomassie Brilliant Blue R-250.

### Western Blotting

For Western blot analysis, proteins were transferred to PVDF membranes (Millipore) and stained with Ponceau S to monitor protein transfer. Membranes were blocked overnight at 4°C with TBS-T (10 mM Tris–HCl, 150 mM NaCl, pH 7.5 with 0.1% Tween 20) containing 5% (w/v) skimmed milk. Thereafter, the membranes were incubated overnight at 4°C with the primary antibody used in TBS-T containing 1% (w/v) skimmed milk. Antibodies used were: anti-human TTR polyclonal antibody (Santa Cruz Biotechnology) at a dilution of 1∶5000; anti-argpyrimidine (Jaica) at a dilution of 1∶2000; anti-human soluble RAGE (Santa Cruz Biotechnology) at a dilution of 1∶5000; anti-fibrinogen (Calbiochem) at a dilution of 1∶10000. Membranes were washed three times for 10 min each with TBS and incubated for 1 h at room temperature with anti-rabbit IgG (Roche) (at 1∶10000 dilution) or anti-mouse IgG (Roche) (at 1∶10000 dilution). Immunoreactivity was detected with diaminobenzidine as a chromomeric substrate, following the manufacturer's instructions (Pierce). Each dried blot was scanned at 600 dpi, saved as a TIFF file and analysed using the “Gel Analysis” functions of *ImageJ* program (National Institutes of Health, available at http://rsb.info.nih.gov/ij/). Background correction was done using a “rolling ball” method with a radius of 4 times the width of a band. Result of the analysis is a value for each band which is proportional to the Integrated Density Value (IDV) of that band [33].

### In Gel Protein Digestion

Protein bands were manually excised from the gels and digested using trypsin as described [33], [34], [35]. Briefly gel protein bands were washed in MilliQ water and distained with 50% (v/v) acetonitrile and subsequently with 100% acetonitrile. Cysteine residues were reduced with 10 mM DTT and alkylated with 50 mM iodoacetamide. Gel pieces were dried by centrifugation under vacuum and rehydrated in digestion buffer containing 50 mM NH_4_HCO_3_ and 6.7 ng.µL^−1^ of trypsin (modified porcine trypsin, proteomics grade, Promega) at 4°C. After 30 min, the supernatant was removed and discarded and 20 µL of 50 mM NH_4_HCO_3_ were added. Digestions were allowed to proceed at 37°C overnight (16–18 hours). After digestion, the remaining supernatant was removed and stored at −20°C.

### Mass Spectrometry

For protein identification and glycation assignment, a MALDI-TOF/TOF 4800 Plus mass spectrometer (Applied Biosystems) was used. Desalting and concentration of tryptic peptides was carried out with in-house made chromatographic microcolumns using GELoader tips packed with POROS R2 (Applied Biosystems). Peptides were directly eluted from the microcolumns onto the MALDI plate using α-ciano-4-hydroxycinnamic acid (5 mg.ml^−1^) in 50% (v/v) acetonitrile with 0.1% (v/v) formic acid as matrix. MS Experiments were performed in positive reflectron mode for monoisotopic peptide mass determination. The mass spectrometer was externally calibrated using des-Arg-Bradykinin (904.468 Da), angiotensin 1 (1296.685 Da), Glu-Fibrinopeptide B (1570.677 Da), ACTH (1–17) (2093.087 Da), and ACTH (18–39) (2465.199) (4700 Calibration Mix, Applied Biosystems). MS Spectra were collected in a result-independent acquisition mode, typically using 1000 laser shots per spectra and a fixed laser intensity of 3000 V. For tandem experiments, fifteen of the most intense precursors were selected for MS/MS, the weakest precursors being fragmented first. MS/MS Analyses were performed using CID (Collision Induced Dissociation) with 1 kV collision energy and 1×10^6^ torr air pressure. 2000 Laser shots were collected for each MS/MS spectrum using a fixed laser intensity of 4000 V. Raw data were generated by the 4000 Series Explorer Software v3.0 RC1 (Applied Biosystems) and tryptic peptide contaminant *m/z* peaks resulting from trypsin auto digestion (842.508 Da; 1045.564 Da; 2211.108 Da; 2225.119 Da) were excluded when generating the peptide mass list used for comparison with the theoretical tryptic digest. Proteins were identified using the GPS explorer software (Applied Biosystem) and identifications were further confirmed using the ProteinPilot software (Applied Biosystem), as previously reported [36]. To identify glycated-modified peptides and amino acid residues, an inclusion list with predicted monoisotopic masses for modified peptides of the identified proteins were loaded in the tandem MS mode. Confirmation of the presence of glycated peptides by mass spectrometry was performed as previously described [37], [38]. Briefly, as protein glycation modifies lysine and arginine side chains it causes misscleaves when hydrolysing proteins with trypsin. If a lysine or an arginine residue is modified, it is possible to predict the mass increase associated to any given known MAGE in a peptide with a misscleavage. The relative quantitation of TTR variants in heterozygotic subjects by mass spectrometry was performed as previously described [39], [40], briefly tryptic peptides were desalted and concentrated using Poros reverse phase R2 (Applied Biosystems) and eluted directly to the MALDI target AnchorChip (Bruker Daltonics, Bremen) with a matrix solution of α-cyano-4-hydroxycinnamic acid (Fluka) prepared at a concentration of 10 µg.µL^−1^ in 50% (v/v) acetonitrile with 0.1% (v/v) trifluroacetic acid. Peptide mixtures were analyzed by MALDI-FTICR-MS in a Bruker Apex Ultra, Apollo II Combi-Source (Bruker Daltonics), with a 7 Tesla magnet (Magnex Corporation). Monoisotopic peptide masses were determined using the SNAP 2 algorithm in Data Analysis software version 4.0 (Bruker Daltonics). External calibration was performed by using bovine serum albumin tryptic digest spectrum and the data processed and analyzed with Biotools 3.2 (Bruker Daltonics).

### TTR-GST Pull Down assay in plasma

Human TTR cloned in p426GPD plasmid (a kind gift from Tiago Outeiro, Instituto de Medicina Molecular da Universidade de Lisboa, Portugal) was cleaved with the restriction enzymes *Bam*HI and *Xho*I. The inserts were purified after electrophoresis separation on 1% (w/v) agarose gel in TBE buffer. The expression vector pGEX 4T.2-TTR was obtained by ligation of the inserts with the expression vector pGEX 4T.2 (Amersham biosciences), which was doubly digested with *Bam*HI and *Xho*I. The vector pGEX 4T.2-TTR was transformed into *E. coli* DH5α and purified using the High Pure Plasmid Isolation Kit (Roche). For the recombinant protein expression and purification, the resulting construct was transformed into *E. coli* BL21^+^ by heat shock and a single colony was used to inoculate 10 mL of Luria Broth medium supplemented with ampicillin (50 µg.mL^−1^) at 37°C with shaking. The culture was grown overnight and 10 mL were used to inoculate 1 L of LB medium at 37°C. Protein expression was induced with 1 mM IPTG (isopropyl-β-D-thiogalactopyranoside) when the absorbance at 600 nm reached 0.4. After 3 hours of growth, cells were immediately chilled on ice and harvested by centrifugation at 15000 *g* for 20 min at 4°C. Cells were frozen overnight at −80°C and afterwards the cell pellet was suspended in 10 mL phosphate-buffered saline (PBS), pH 7.4, sonicated 10 times for 30 s and centrifuged for 20 min at 14000 *g* at 4°C. The supernatant was stored at −80°C until use. TTR in fusion with glutathione S-transferase (GST) (pGEX 4T.2-TTR) and GST alone (pGEX 4T.2) were purified with gluthatione sepharose 4BT beads (Amersham). For the pull-down assay, the recombinant proteins and plasma (diluted 1∶2 in incubation buffer, 100 mM potassium acetate, 30 mM Hepes-KOH, pH 7.5, supplemented with 4 mM of leupeptine, aprotinine and PMSF protease inhibitors) were incubated with rotation at 4°C for 3 hours and washed four times with washing buffer (100 mM potassium acetate, 30 mM Hepes-KOH at pH 7.5, 2 mM of leupeptine, aprotinine and PMSF). Plasma was diluted 1∶2 in washing buffer before incubation. After washing, bound proteins were eluted from the beads with denaturating buffer (62.5 mM Tris-HCl, pH 6.8, 20% (v/v) glycerol, 2% (w/v) SDS, 5% (v/v) β-mercaptoetanol) and boiled for 5 min. Eluted proteins were separated by SDS-PAGE using 12% resolving gels, which were silver stained using a silver staining kit (GE Healthcare). Each pull down assay was repeated at least three times.

### Methylglyoxal preparation

High purity methylglyoxal was prepared by fractional distillation under reduced pressure in nitrogen atmosphere as described [41]. Once prepared, methylglyoxal solutions were standardized by enzymatic assay with glyoxalase I and II. Purity was verified by HPLC analysis and ^13^C NMR (Bruker advance 400 MHz).

### Glycation in vitro of α-crystallin and human fibrinogen by methylglyoxal

Bovine lens α-crystallin (Sigma) and human whole fibrinogen (Calbiochem) (10 mg.ml^−1^) in 0.1 M sodium phosphate buffer (pH 7.4) were incubated with 2.5 and 10 mM of methylglyoxal in sterile conditions for 7 days at 37°C. Following incubations, all samples were dialyzed against 0.1 M sodium phosphate buffer (pH 7.4) for 48 hours at 4°C.

### Chaperone activity assay of α-crystallin and human fibrinogen

Chaperone activity of α-crystallin and human fibrinogen were assayed in a Beckman DU 7400 spectrophotometer. Total reaction volume was 2.5 ml. Five milligrams of insulin or two milligrams of lysozyme (Sigma) were incubated with 2 mM DTT in 0.1 phosphate buffer (pH 7.0) containing 2 mM EDTA at 37°C. Protein aggregation was followed by the absorption increase due to increased light scattering in time, monitored at 360 nm for 60 min at 37°C. DTT is a commonly used aggregation induction chemical agent. By reducing sulfhydryl bonds, proteins adopt a linear conformation and become prone to aggregation, most likely due to exposure of hydrophobic regions and the onset of nucleation centres [42]. To assay chaperone activity, glycated and non-glycated α-crystallin and human fibrinogen were added to the mixture at twice the molar concentration of the target protein.

## Results

### Decreased TTR stability in FAP patients

**Figure 1:**
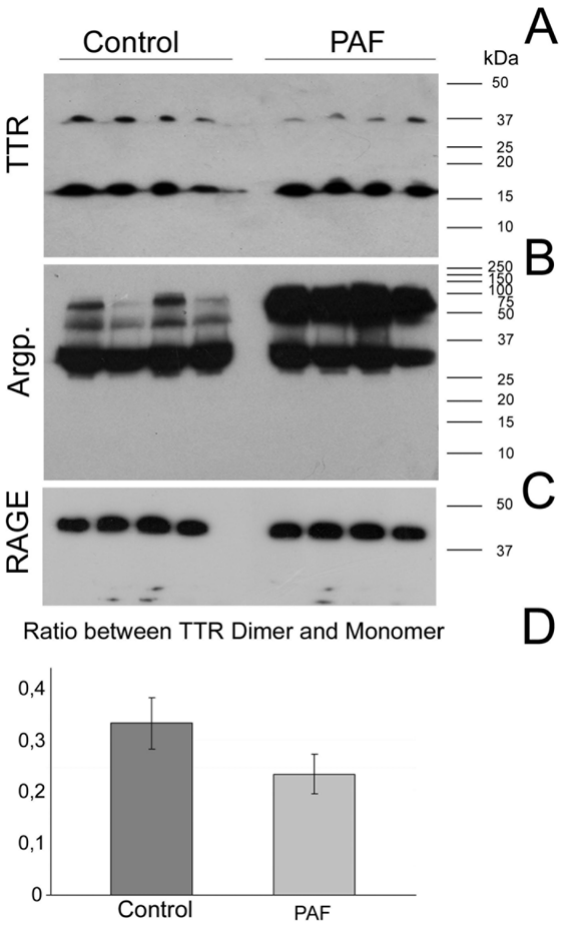
Western blot analysis of human plasma proteins. Samples from four control and four FAP individuals were analysed to detect (A) TTR; (B) Argpyrimidine-modified proteins and (C) soluble RAGE; (D) Ratio between the intensity of TTR dimer and monomer. The two-tailed P value equals 0.0253. This difference is statistically significant with a 95% confidence interval.

TTR is a tetramer composed of two dimers that bind to each other and due to the large difference in the number of hydrophilic interactions, the monomer-monomer contacts within the dimer are more stable than the dimer-dimer interactions [43]. TTR tetramer and dimer dissociation are essential steps towards aggregation and amyloid fibril formation. Therefore, we analysed the relative amounts of TTR monomers and dimers in FAP and control subjects by SDS-PAGE, as previously used to evaluate the relative amount of TTR dimer to monomer species [44]. Also, the stable sample-specific dimer/monomer ratios, achieved under conditions of partial dimer to monomer transition, reflect the conformational monomer stability relative to the dimeric form [45]. Once a dimer dissociates, the monomers are captured in a complex with SDS and their ability to associate as a dimer is compromised. After SDS-PAGE separation, under conditions of partial dimer to monomer transition, TTR from control and FAP groups was detected by Western blot, allowing the observation of both dimer and monomer and also to perform a relative quantification (Figure 1A). Monomer and dimer area signals were quantified using ImageJ software. The signal intensity of each protein band was normalized using the sRAGE protein band signal (Figure 1C), for each lane, as loading control. We observed that FAP individuals present a significantly lower ratio of dimer/monomer than control individuals (Figure 1D, t-test for the significance of the difference between the means of two independent samples; p = 0.0253). This observation is in agreement with *in vitro* models of FAP amyloidosis, which revealed a positive relationship between TTR amyloidogenic potential due to a mutation and tetramer stability decrease [6].

### Differential plasma protein glycation in FAP patients

We previously discovered that argpyrimidine is present in amyloid fibrils from Portuguese type FAP patients [22], being the first evidence that methylglyoxal-protein glycation is involved in FAP and that glycation might play an important role in this pathology. In this work, we compared the glycated plasma proteomes from control subjects and FAP patients, targeting argpyrimidine as a specific marker of methylglyoxal-derived glycation. Plasma proteins from control and FAP individuals were separated by SDS-PAGE and argpyrimidine-modified proteins were detected by Western blot with a specific antibody against this MAGE (Figure 1B). Although glycated proteins were found in control and FAP subjects, in the latter case, a higher level of argpyrimidine modified proteins are present in comparison to control subjects. This is particularly noticeable in a protein band with a molecular mass of 80 kDa which shows a strong signal for argpyrimidine only in FAP individuals and corresponds to a faint protein band in control subjects (Figure 1B). It is noteworthy that glycated proteins present a similar pattern in all four controls and FAP individuals, showing a high homogeneity and specificity of plasma protein glycation even in different individuals.

**Figure 2:**
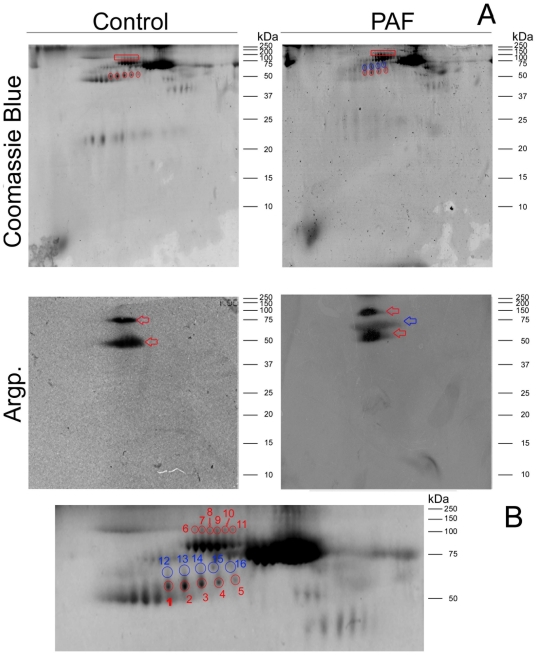
Two dimensional electrophoresis of control and FAP plasma proteins stained with Coomasie and Western blot analysis of argpyrmidine modified proteins. (A) Full gel image; (B) Zoom image of the gel area at which protein spots were removed. Assigned protein spots were excised for protein identifications (Table 1). Red marks correspond to common glycated proteins and blue marks correspond to protein glycated differentially in FAP individuals.

To identify these argpyrimidine modified proteins, plasma proteins were separated by 2D-PAGE and glycated proteins were detected by Western blot as before (Figure 2A). In red (spots 1–11) we assigned glycated proteins found in both control and FAP individuals, whereas blue (spots 12–16) shows assigned glycated proteins found exclusively in FAP patients. The molecular mass of these proteins spots is similar to the protein bands detected by Western blot for argpyrimidine in one-dimensional electrophoresis and most likely corresponds to multiple forms of the same protein. All assigned protein spots were excised from Coomassie stained two dimensional gels (Figure 2B), trypsin digested and proteins identified by MALDI-TOF-TOF-MS using tandem MS data. All identified proteins are reported in Table 1. The differentially argpyrimidine glycated proteins in FAP individuals were unequivocally identified as several isoforms of fibrinogen and immunoglobulins. The glycation of these identified proteins was further confirmed by tandem MS data. As previously reported by our group, MS spectra of tryptic digested glycated proteins contains information regarding the nature and location of the glycated amino acid residue [38]. Since only arginine residues are modified by methylglyoxal with the formation of argpyrimidine, tryptic digestion of glycated proteins produces peptides with an arginine misscleavage associated with an 80 Da mass increase, characteristic of an argpyrimidine modification. To identify the glycated peptides, protein spots corresponding to the differentially glycated proteins were removed from the 2D-PAGE gels of FAP patients plasma, digested with trypsin and analysed by MS. Proteins from control individuals 2D gels spots, matching the glycated proteins spots in the FAP individuals 2D gels, were also removed and the tryptic digested protein mass spectra were acquired and compared. A peptide was considered to be glycated only if it was absent from the MS spectra of the control sample. From this analysis, it was possible to observe several glycated peptides of the identified proteins, absence from the corresponding protein spot removed from control individuals, thus confirming, at a molecular level, that glycation occurred and argpyrimidine was present.

**Table 1:** Identification of argpyrimidine-modified proteins in human plasma.

Spot	Identification	Accession code	Protein Score	Protein Score C. I. %	Total Ion Score	Total IonC. I. %	Peptides Matched
1	Fibrinogen beta chain	FIBB_HUMAN	619	100	469	100	19
2	Fibrinogen beta chain	FIBB_HUMAN	628	100	467	100	20
3	Fibrinogen beta chain	FIBB_HUMAN	899	100	711	100	22
4	Fibrinogen beta chain	FIBB_HUMAN	892	100	676	100	22
5	Fibrinogen beta chain	FIBB_HUMAN	642	100	449	100	20
6	Ig gamma-1 chain C region	IGHG1_HUMAN	595	100	528	100	17
	Ig gamma-3 chain C region	IGHG3_HUMAN	218	100	189	100	7
7	Ig gamma-1 chain C region	IGHG1_HUMAN	468	100	380	100	13
8	Ig gamma-1 chain C region	IGHG1_HUMAN	606	100	539	100	16
	Ig gamma-1 chain C region	IGHG1_HUMAN	576	100	477	100	16
9	Ig gamma-1 chain C region	IGHG1_HUMAN	586	100	481	100	16
	Ig heavy chain V-III region TIL	HV304_HUMAN	109	100	83	100	6
10	Ig gamma-1 chain C region	IGHG1_HUMAN	612	100	511	100	13
11	Ig gamma-1 chain C region	IGHG1_HUMAN	610	100	514	100	11
**12**	Fibrinogen beta chain	FIBB_HUMAN	790	100	586	100	12
**13**	Fibrinogen beta chain	FIBB_HUMAN	644	100	507	100	9
**14**	Fibrinogen alpha chain	FIBA_HUMAN	309	100	168	100	7
**15**	Ig alpha-1 chain C region	IGHA1_HUMAN	402	100	358	100	8
**16**	Ig alpha-2 chain C region	IGHA2_HUMAN	221	100	191	100	5

Spot numbers correspond to the ones assigned in Figure 2B.

To investigate if the higher level of protein glycation observed in FAP patients were related to any diabetes risk factors, we analysed the soluble forms of the RAGE protein (sRAGE) by Western blot. An association between the circulating sRAGE levels and diabetes was previously observed [46] and this condition that lead to an accumulation of glycation adducts. In our case, no differences were observed in sRAGE levels between the control and FAP patients, in agreement with the lack of diabetes background in FAP individuals.

### Plasma protein glycation increases and TTR stability decreases over time in DLT individuals

**Figure 3:**
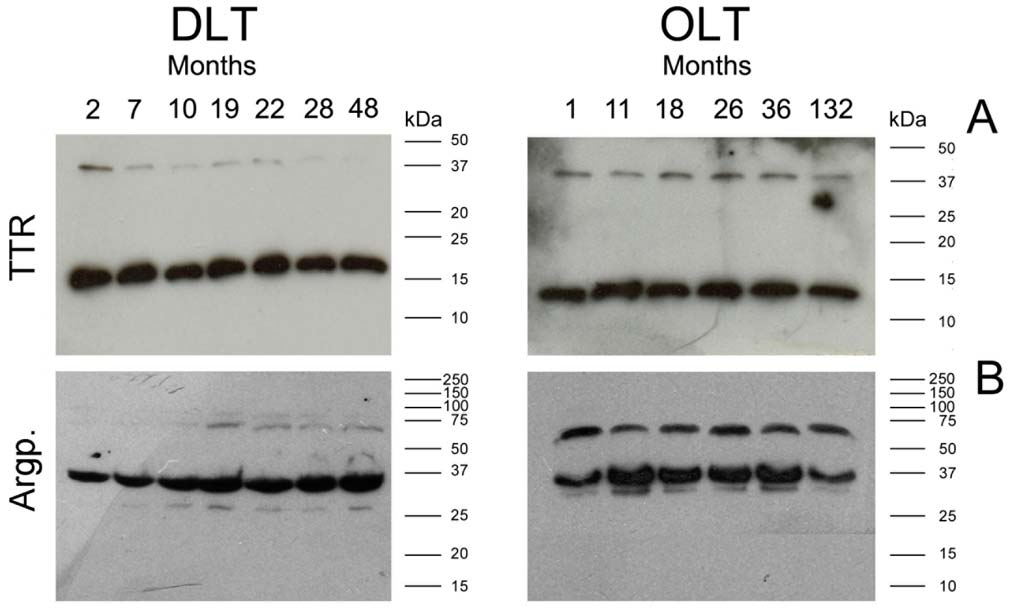
Time course analysis by Western blot of TTR and argpyrimidine modified proteins after liver transplantation. (A) Western blot analysis of TTR in the plasma from DLT individuals over time (from 2 months to 4 years) and from OLT individuals over time (from 1 month to 11 years); (B) Western blot analysis of argpyrimidine modified proteins in the plasma from DLT individuals over time and from OLT individuals over time.

Since plasma TTR is produced by the liver, disease progression can be halted by OLT from cadaveric donors, leading to the clearance of mutated TTR from the plasma of a FAP transplanted recipient [47]. As the livers from FAP patients undergoing OLT are functionally normal, except for the production of a mutated TTR variant, the domino liver transplantation (DLT), in which FAP livers are transplanted to patients with liver failure, was recently introduced. Thus, DLT replaces non-mutated TTR by the amyloidogenic TTR variant V30M. As the age of disease onset in FAP TTRV30M ranges from the late 20s to the early 40s, it was expected that livers explanted from FAP patients would function in recipients without formation of amyloid fibrils for a long period of time. However, at least two recent reports indicate that amyloid deposition or FAP symptoms appeared in domino recipients much sooner than expected [48], [49]. To investigate a possible role of protein glycation in this rapid disease progression, we monitored TTR dimer/monomer ratios and the glycation levels of samples from individuals that were subjected to OLT and DLT, from 1 month to 11 years (OLT) and from 2 months to 4 years (DLT) after liver transplantation. As shown in Figure 3A, TTR dimer maintains its stability over time in FAP individuals that were subjected to OLT, for that moment on having only non-mutated TTR in circulation. Likewise, there is no change over time both in the number and relative amount of glycated plasma proteins after liver transplantation (Figure 3A). The opposite was observed in individuals subjected to DLT. As shown in Figure 3B, the glycation of plasma proteins increases over time after DLT. In addition, for the same time lapse we observed a marked reduction of TTR dimer stability in these individuals. Thus, a relationship between a reduction of the TTR dimer stability and an increase in the glycation levels is apparent in patients subjected to DLT. In this work, all TTR mutation carrying individuals or patients subjected to DLT are heterozygous, meaning that the liver expresses both the mutated (V30M) and the native TTR form. To exclude the hypothesis that the reduced TTR dimer stability could be due to the different amounts of the two TTR forms (native and V30M) expressed over time, we determined the WT to V30M ratio by MALDI–FTICR-MS, as previously described by us [39], [40]. No significant differences were observed between the ratios of the two TTR forms. As expected, this ratio only shows slight differences between different individuals (Table S1, Figure S1).

### Fibrinogen is a TTR protein interaction partner in plasma

**Figure 4:**
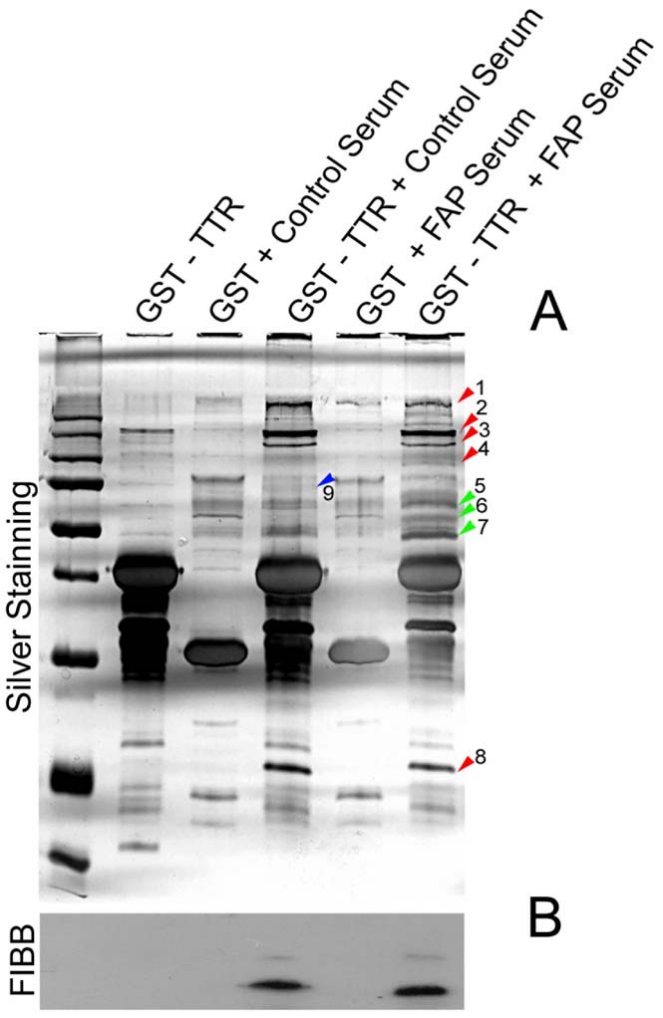
TTR Protein binding assay in human plasma. (A) - TTR was expressed in bacteria as GST fusion protein. After purification GST alone and GST in fusion with TTR (GST-TTR) were incubated with plasma, affinity purified with gluthathione beads and analysed by SDS-PAGE. Purified fusion protein without plasma incubation (GST-TTR), GST alone incubated with plasma (GST+Control Plasma for incubation with plasma from control individuals and GST+FAP Plasma for incubation with plasma from FAP individuals) were also analysed as controls. Assigned protein bands were excised for identification (Table 2). (B) Western blot analysis of fibrinogen after the pull down assay.

To uncover the transthyretin protein interaction network in plasma, TTR affinity purification assays were used to identify its putative protein binding partners. For these assays, TTR was expressed in bacteria as a GST fusion product, purified and used as bait for pull down assays from control and FAP plasma. As negative control, GST was expressed in bacteria, purified and used as bait. The proteins specifically eluting with the TTR tagged protein were identified by MS/MS peptide mapping using MALDI TOF-TOF MS. As depicted in Figure 4A, nine proteins were identified as specifically eluting with the TTR tagged protein: five were co-purified with TTR from both control and FAP plasma (proteins bands 1, 2, 3, 4 and 8, labelled with red arrows), three were co-purified only from FAP plasma (protein bands 5, 6 and 7, labelled with green arrows) and one was purified only from control plasma (protein band 9, labelled with blue arrow). The retinol binding protein (RBP) was identified in co-elutions from both control subjects and FAP patients, validating the pull down experiment since RBP is a well characterized TTR binding partner in plasma and the cephalorachidian fluid (Figure 4A, protein band 8). Several proteins were identified as TTR interacting partners in plasma, namely albumin, immunoglobulins and cis-trans isomerase (Table 2). Among these, fibrinogen deserves a closer look not only because of its recently discovered chaperone activity [50] but also due to its differential glycation pattern that distinguishes control subjects from FAP patients. To further validate this result, the affinity purification assay was repeated and fibrinogen beta chain was detected by Western blot (Figure 4B). Fibrinogen was unequivocally purified by TTR tagged protein pull-down assay from both control and FAP patients. Considering the fact that protein glycation by methylglyoxal is known to exert effects on the activity of chaperone proteins we speculated that this could also be the case of fibrinogen and as such to be related with TTR stability and the molecular disease mechanisms of FAP.

**Table 2:** Identification of TTR interacting protein partners in human plasma.

Band	Identification	Accession code	Protein Score	Protein Score C. I. %	Total Ion Score	Total Ion C. I. %	Peptides Matched
1	Fibrinogen beta chain	FIBB_HUMAN	1250	100	1003	100	24
2	Fibrinogen beta chain	FIBB_HUMAN	102	100	85	100	6
3	Fibrinogen gamma chain	FIBG_HUMAN	1050	100	864	100	23
4	Fibrinogen alpha chain	FIBA_HUMAN	1280	100	1227	100	25
5	Immunoglobulin mu-chain D-J4-region	IGHM_HUMAN	630	100	542	100	20
6	Ig heavy chain V region M603	HVM20	157	100	150	100	8
7	Peptidyl-prolyl cis-trans isomerase	Q13427	436	100	377	100	14
8	Retinol binding protein	RET4	675	100	595	100	19
9	Serum albumin	ALB	130	100	39	85	17

Protein band numbers are the ones assigned in Figure 4.

### Fibrinogen prevents plasma TTR thermal-induced protein aggregation and glycation decreases it chaperone activity

**Figure 5:**
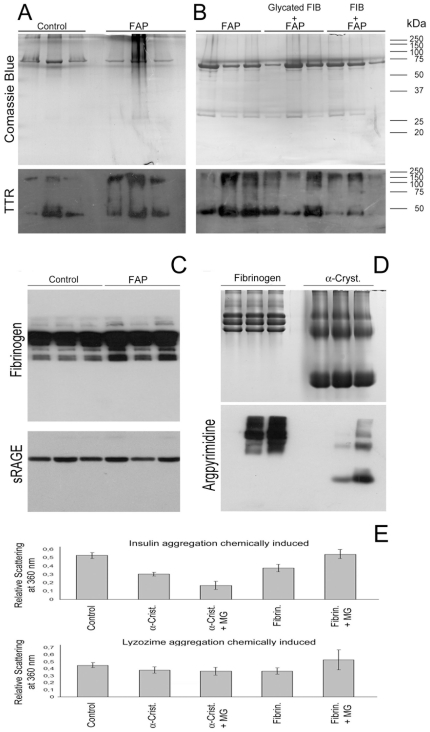
Fibrinogen prevents plasma TTR thermal-induced protein aggregation and glycation decreases it chaperone activity. (A) Western blot analysis of TTR in the plasma from three control and three FAP individuals after aggregation induced by temperature. (B) Western blot analysis of TTR in the plasma from three FAP individuals after aggregation induced by temperature in the absence of fibrinogen, in the presence of glycated fibrinogen and in the presence of non-glycated fibrinogen. (C) Western blot analysis of fibrinogen in the plasma from three control and three FAP individuals, Western blot of sRAGE was used as loading control. (D) Coomassie blue stained gel of α-crystallin and fibrinogen without glycation and glycated with 2.5 and 10 mM of methylglyoxal. Western blot analysis of argpyrimidine modified proteins. (E) Chemically (DTT) induced protein aggregation of insulin and lysozyme (The aggregation of insulin and lysozyme alone, respectively, are used as controls) in the presence of α-crystallin and fibrinogen with and without glycation. Error bars indicate standard deviation.

The ability of whole fibrinogen to inhibit the thermal-induced TTR aggregation was analyzed using plasma from healthy and FAP subjects. After incubation at 43°C for 48 h, a substantially larger amount of TTR precipitate was found in the plasma of FAP individuals when compared to plasma of control individuals (Figure 5A). Moreover not only the total amount of TTR is higher, but also the amount of aggregated TTR (high molecular mass protein bands) is also greater in FAP individuals. This precipitation could be rescued by adding exogenous fibrinogen (final concentration of 2.8 µg.mL^−1^) into the plasma of FAP individuals before incubation (Figure 5B). However, the addition of glycated fibrinogen to plasma does not rescue TTR (Figure 5B). This result indicates that fibrinogen's glycation by methylglyoxal impairs its ability to inhibit TTR aggregation. Considering the higher glycation levels of fibrinogen in FAP patients, this might have an important impact in disease progression.

To evaluate the effects of glycation on fibrinogen's chaperone activity, we incubated human fibrinogen with high purity methylglyoxal, prepared in house as described, using 2.5 and 10 mM. Glycation by methylglyoxal was probed by Western blot, showing as expected a dose-dependent glycation pattern (Figure 5D). Since argpyrimidine modification of α-crystallin has been described as largely responsible for its increased chaperone function [49], this protein was also glycated *in vitro* and used as a positive control. We then compared the ability of glycated and non-glycated lens α-crystallin to prevent chemically induced protein aggregation of two target proteins, insulin and lysozyme, commonly used as model proteins for chaperone activity studies. In agreement to previous reports, argpyrimidine modification increased α-crystallin's chaperone activity by nearly 2-fold, reducing insulin aggregation (Figure 5E), Similar results were obtained with lysozyme, confirming the increased chaperone activity of α-crystallin upon glycation by methylglyoxal (Figure 5E). As shown in Figure 5E, insulin and lysozyme aggregation were significantly suppressed by fibrinogen (t-test for the significance of the difference between the means of two independent samples; p = 0.011 for insulin and p = 0.061 for lysozyme. Student's-t test with a 90% confidence interval. P≤0.1 was considered statistically significant). However, glycation by methylglyoxal significantly reduces fibrinogen chaperone activity when both insulin and lysozyme were used as target proteins, there is no statistical significant diference between aggregation of insulin and lysozyme alone (control) and in the presence of Glycated fibrinogen (Fibrinogen+MG).

Interestingly, human fibrinogen presents an increased expression in FAP patients, as shown in Figure 5C. Higher levels of this protein were also observed in other amyloid disorders, such as Alzheimer and vascular dementia [51].

## Discussion

In this work, we discovered that human plasma proteins are differentially glycated in FAP patients in comparison with healthy control subjects. Moreover, after DLT these glycated proteins increase its abundance over time. We have also discovered that fibrinogen, one of the differentially glycated proteins, is a TTR interacting partner in plasma. Furthermore, it was recently discovered that fibrinogen has chaperone activity and we demonstrated in this work that upon glycation by methylglyoxal its chaperone activity decreases. Taken together, these observations lead us to propose that fibrinogen glycation *in vivo* reduces its chaperone activity, compromising TTR tetramer stability and contributing to disease.

Although all FAP patients have identical concentrations of TTR V30M in plasma and cerebrospinal fluid, age at onset varies widely between 20 and 70 years. Therefore, despite the identification of more than 80 TTR point mutations associated with FAP, the process of fibril formation and its relationship with pathogenesis remains unclear. Formation of amyloid fibrils by non-mutated native TTR, as in senile systemic amyloidosis (SSA) [7] implies that other factors besides genetic determinants must be considered in FAP's pathogenesis. Since the first symptoms in FAP appear much earlier than in SSA, TTR point mutations only seem to accelerate fibril formation by increasing the intrinsic TTR amyloidogenicity.

The identification of glycation adducts in FAP patients was previously described in long lived avascular tissues with low turnover, like vitreous bodies and amyloid fibrils, being suggested that these tissues were more prone to undergo glycation [52]. In this work, we evaluated the presence of MAGE-modified proteins in the plasma of FAP patients, relatively to healthy control individuals. We identified two proteins differentially glycated by argpyrimidine in FAP individuals. These results are surprising since protein glycation is commonly associated to hyperglycaemia conditions and FAP patients do not present a disturbed metabolism of glucose, actually showing hypoglycaemia after transient hyperinsulinemia upon oral administration of a loading dose of glucose [53]. Furthermore, FAP patients show transient hypoglycaemia during their daily life [53]. In addition, no differences were detected in sRAGE levels between control and FAP subjects. Protein glycation is often associated with crosslink formation, matrix dysfunction, reduced protein solubility and increased protease resistance, implicated in the pathophysiology of normal ageing and in the pathogenesis of the long-term clinical complications of diabetes [54], [55], [56]. The formation of glycation adducts depends on the plasma concentration of glucose, methylglyoxal and other glycation agents [56] and these compounds are strongly associated with the severity of diabetes complications, particularly cardiovascular and renal ones. We propose that the increased levels of methylglyoxal glycation found in FAP might be related to renal failure. Indeed, in renal failure, plasma and tissue levels of glycation adduct are completely independent of plasma glucose [55]. FAP patients present low levels of erythropoietin (EpoI), suggesting that these abnormalities could be related to the amyloid deposits in renal interstitium [57]. Renal disease in FAP patients ranges from proteinuria to end-stage renal failure, with replacement of renal function by dialysis. In comparison to FAP patients with normal renal function, the progression of the neurologic disease is delayed in FAP patients enduring dialysis [58]. However, hemodialysis and hemodiafiltration are ineffective in removing TTR, in spite of the lower stability of the TTR V30M variant. Since the protective feature of hemodialysis on the progression of amyloidosis is not due to the clearance of this abnormal protein from plasma [58], we suggest that plasma glycation agents are removed by hemodialysis and so their contribution to FAP decreases after this procedure. It might be interesting to consider other glycation agents as well, particularly fructose, more reactive than glucose and whose plasma concentration is also dependent on dietary habits. Different dietary habits have an impact on glycation and may also contribute to explain individual differences [59].

Human fibrinogen specifically interacts with and suppresses aggregation of a wide spectrum of stressed proteins [50]. It was previously observed that human fibrinogen can inhibit the formation of Sup35 fibril, which shares key features of amyloid fibrils of mammalian prions and amyloid proteins [60] indicating a potential role of fibrinogen on misfolding diseases as a molecular chaperone. It was suggested that fibrinogen may interact with prefibrillar species during the fibril formation, redirecting the aggregation process. It is also noteworthy that high levels of fibrinogen are found in both Alzheimer's disease and vascular dementia [51]. In this work, we observed that fibrinogen expression is increased in FAP patients. It is highly likely that increased levels of fibrinogen in patients with different pathologies that share common molecular mechanisms may be a response to the increased need of extracellular chaperone activity under such pathological conditions and they are aggravated by fibrinogen glycation which decreases its chaperone activity, as discovered in this work.

**Figure 6:**
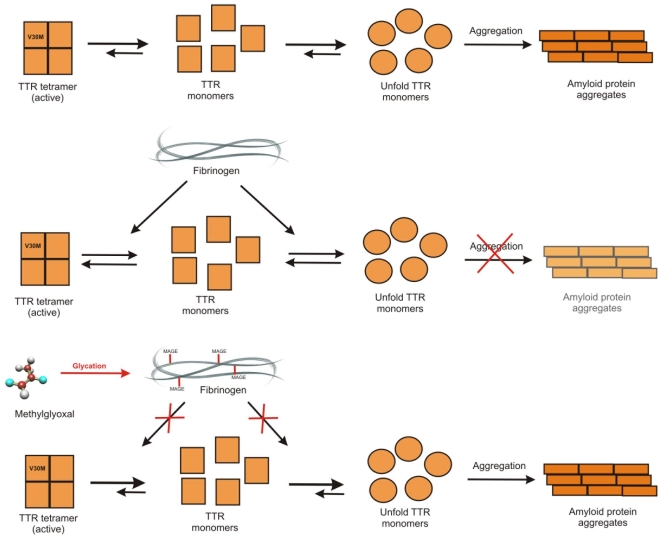
Molecular model of the effects of fibrinogen glycation *in vivo* in FAP. *In vitro* studies established that amyloidogenic TTR variants (as V30M) have a higher tendency to follow an aggregation pathway high the formation of amyloid deposits. Considering our findings that fibrinogen interacts with TTR in the plasma, we proposed that fibrinogen chaperone activity reduces TTR propensity towards fibril formation. However, the specific glycation of fibrinogen compromises its vital chaperone activity and in these conditions TTR becomes more prone to aggregation.

It is well known that protein structure and biological function are altered upon glycation [56]. Considering our finding that fibrinogen interacts with TTR in the plasma, we propose a new molecular model for TTR aggregation that the fibrinogen chaperone activity reduces TTR propensity towards fibril formation. When fibrinogen is glycated, this function is compromised and TTR becomes more prone to aggregation (Figure 6). A molecular explanation on why plasma fibrinogen is more prone to modification by methylglyoxal is not yet possible. Nevertheless, studies with yeast cells show that among all its expressed proteins, enolase (eno2p) is the most relevant glycation target [37]. Besides eno2p being an abundant protein, arginine glycation sites were identified in arginine rich clusters that might favor glycation by methylglyoxal. The structure of fibrinogen was recently made available and several potential arginine rich regions are apparent [61]. Nevertheless, this is a matter for further research.

Although liver transplantation has become a well-established treatment for halting the progression of FAP-related clinical symptoms, no truly effective therapy has been designed [62], [63], [64] and several problems have arisen from this procedure [65], [66]. Timing for liver transplantation in still one of the main problems associated to this therapy. First, FAP gene carriers who have no clinical symptoms cannot undergo liver transplantation before the onset of the disease. Second, FAP's clinical complications present before the surgery will remain unchanged after liver transplantation. Protein glycation in FAP may thus be an important biomarker long before symptoms appear.

Finally, the effects of glycation on chaperone activity in connection with conformational diseases are likely to be of high relevance for the understanding of the biochemical mechanisms underlying these pathologies and may offer pathways towards the discovery of novel and effective therapeutic opportunities on conformational diseases.

## Supporting Information

Figure S1 **Peptide mass Fingerprint of the TTR protein band after tryptic digest in transplanted individuals over time.** A – The ion 1398,7 is present only in individuals that carry the TTR V30M, and so FAP individuals and transplanted with sequential liver and. This ion corresponds to the peptide with the sequence GSPAINVAMHVFR of the TTR V30M. B - The ion 1366.8 is present both in control and FAP individuals and also in individuals transplanted with cavaderic liver and sequential liver. This ion corresponds to the peptide with the sequence GSPAINVAVHVFR of the TTR WT.
(TIF)

Table S1 **Relative quantification of TTR in serum from Sequential transplanted individuals and orthotic transplanted individuals, using peak 1394.732 as internal standard (each value corresponds to the average of three spectra acquired).**
(DOC)
