# Supplementary material for: Beyond Genetic Factors in Familial Amyloidotic Polyneuropathy: Protein Glycation and the Loss of Fibrinogen's Chaperone Activity
Source: PLoS One. 2011 Oct 28;6(10):e24850. doi: 10.1371/journal.pone.0024850 (PMC3203866; doi:10.1371/journal.pone.0024850)
Supplement: Table S1 — Relative quantification of TTR in serum from Sequential transplanted individuals and orthotic transplanted individuals, using peak 1394.732 as internal standard (each value corresponds to the average of three spectra acquired). (DOC) [file pone.0024850.s002.doc]

Table S1 - Relative quantification of TTR in serum

| Individual | Mass | | ratio | Relative quantity |
| --- | --- | --- | --- | --- |
|  | 1366.76 | 1394.62 |  |  |
| OLT 1 month | 2050000 | 6000000 | 0.34 | 2.36  (The two-tailed P value is less than 0.0001) |
| OLT 11 months | 3700000 | 11400000 | 0.32 |  |
| OLT 18 months | 1200000 | 3400000 | 0.35 |  |
| OLT 26 months | 3000000 | 8900000 | 0.34 |  |
| OLT 36 month | 2200000 | 6400000 | 0.34 |  |
| OLT 132 month | 1500000 | 4200000 | 0.36 |  |
| DLT 2 months | 510000 | 3700000 | 0.14 |  |
| DLT 7 months | 1510000 | 9000000 | 0.17 |  |
| DLT 10 months | 1730000 | 11000000 | 0.16 |  |
| DLT 19 months | 315000 | 2500000 | 0.13 |  |
| DLT 22 months | 2190000 | 15500000 | 0.14 |  |
| DLT 24 months | 860000 | 6500000 | 0.13 |  |
| DLT 48 months | 865000 | 6000000 | 0.14 |  |
